# Draft Genome Sequence of Mycobacterium hippocampi DL, Isolated from European Sea Bass (Dicentrarchus labrax)

**DOI:** 10.1128/MRA.00816-20

**Published:** 2020-09-03

**Authors:** Panagiota Stathopoulou, Elias Asimakis, Yiannis Petropoulos, George Tsiamis

**Affiliations:** a Department of Environmental Engineering, University of Patras, Agrinio, Greece; University of Maryland School of Medicine

## Abstract

Mycobacterium hippocampi is an acid-fast opportunistic pathogen associated with infections in aquatic animals. Here, we report the draft genome sequence of M. hippocampi strain DL, an isolate from cultured European sea bass (Dicentrarchus labrax) associated with systemic symptomatology.

## ANNOUNCEMENT

Mycobacteriosis, or “fish tuberculosis,” is a serious and often lethal disease of fish, affecting a wide range of species globally both in culture and in wild settings (1, 2). The responsible agents, mycobacteria, are aerobic, acid-fast, and nonmotile rods (3). They are considered important fish pathogens and are associated with multiple symptoms, such as uncoordinated swimming, abdominal swelling, weight loss, skin ulceration, and white nodule formation as granulomas in liver, kidney, and spleen (1, 4).

In 2017, abnormal swimming was recorded in cultured sea bass, followed by other symptoms related to mycobacteriosis. In a culture-dependent approach including necropsy, tissue homogenization, decontamination, and inoculation (5), we succeeded in recovering the putative pathogenic agent from the spleen; the microbial culture system was then optimized according to the method described by Balcázar et al. (6). Colonies on Middlebrook 7H10 agar were irregular, rough, and scotochromogenic with orange pigmentation. The optimum growth temperature was 25°C, whereas no growth was observed at 37°C and little growth was observed at 20°C. The isolate was able to grow between pH 6 and pH 9, with the optimum pH being 7.0. Moreover, microaerophilic conditions promoted the growth of the isolate in solid medium. Genomic DNA was extracted from a single colony and grown under optimum conditions, as described by Haught et al. (7). DNA was quantified in triplicate with the Quant-iT double-stranded DNA high-sensitivity assay (Invitrogen) in an Eppendorf AF2200 plate reader. The genomic DNA library was prepared using the Nextera XT library preparation kit (Illumina, San Diego, CA), following the manufacturer’s protocol. The library was quantified using the Kapa Biosystems library quantification kit for Illumina on a Roche LightCycler 96 quantitative PCR system. The library was sequenced with 30-fold coverage on the Illumina HiSeq platform using a 250-bp paired-end protocol, producing 720,754 reads. Reads were adapter trimmed using Trimmomatic v. 0.30, with a sliding window quality score cutoff value of Q15 (8). FastQC v. 0.11.8 was used to check the quality of the validated reads (9). Reads were *de novo* assembled into 50 contigs using SPAdes v. 3.14 (10). The quality of the assembly was evaluated with Quast v. 5.0.2 (11). Taxonomic assignment of the reads was performed using Kraken v. 2.0.8 (12), and the RAST algorithm v. 2.0 (13–15) was used for genome annotation. The assembled draft genome had a length of 6,251,150 bp, with a GC content of 66.7%. The largest contig was 553,185 bp long, the *L*_50_ value was 8, and the *N*_50_ value was 251,948 bp. Most of the reads were classified into the genus *Mycobacterium*. The draft genome contained 5,984 unique predicted genes. Phylogenomic analysis based on the sequences of 52 single-copy genes showed the greatest similarity with the gene sequences of Mycobacterium hippocampi (87.1%). The genome was also checked based on average nucleotide identity (ANI) with FastANI v. 0.1.2 (16). Default parameters were used for all software unless otherwise specified. Genome sequence comparison with type strains revealed the greatest similarity with M. hippocampi (84.2%).

Our results report the genome sequence of M. hippocampi isolated from Dicentrarchus labrax, which may provide significant epidemiological information on nontuberculous mycobacteria in an aquatic environment.

### Data availability.

The whole-genome shotgun project for M. hippocampi has been deposited in DDBJ/ENA/GenBank under the accession number JABFYL000000000, BioProject number PRJNA630865, and BioSample number SAMN14846999. Raw sequencing reads were deposited in the Sequence Read Archive (SRA) under accession number SRP260393. The version described in this paper is version JABFYL000000000.1.
